# Ten commandments for the future of ageing research in the UK: a vision for action

**DOI:** 10.1186/1471-2318-7-10

**Published:** 2007-05-03

**Authors:** Oscar H Franco, Thomas BL Kirkwood, Jonathan R Powell, Michael Catt, James Goodwin, Jose M Ordovas, Frans van der Ouderaa

**Affiliations:** 1 Unilever Corporate Research, Colworth Park, Sharnbrook, Bedfordshire, MK441LQ, UK; 2 Institute for Ageing and Health, Newcastle University, Newcastle upon Tyne, UK; 3 Help the Aged, London, UK; 4 Nutrition and Genomics Laboratory, Jean Mayer-US Department of Agriculture Human Nutrition Research Center on Aging at Tufts University, Boston, MA, USA

## Abstract

Increases in longevity resulting from improvements in health care and living conditions together with a decrease in fertility rates have contributed to a shift towards an aged population profile. For the first time the UK has more people over age 60 than below 16 years of age. The increase in longevity has not been accompanied by an increase in disease-free life expectancy and research into ageing is required to improve the health and quality of life of older people. However, as the House of Lords reported, ageing research in the UK is not adequately structured and a clear vision and plan are urgently required. Hence, with the aim of setting a common vision for action in ageing research in the UK, a 'Spark Workshop' was organised. International experts from different disciplines related to ageing research gathered to share their perspectives and to evaluate the present status of ageing research in the UK. A detailed assessment of potential improvements was conducted and the prospective secondary gains were considered, which were subsequently distilled into a list of 'ten commandments'. We believe that these commandments, if followed, will help to bring about the necessary implementation of an action plan for ageing research in the UK, commensurate with the scale of the challenge, which is to transform the manifold opportunities of increased longevity into actual delivery of a society living not only for longer, but also healthier, wealthier and happier.

## Why ageing research?

Our society is experiencing unprecedented demographic change [1]. Improvements in health care and living conditions together with a decrease in fertility rates, have contributed to the ageing of the population and a severe demographic redistribution [1,2]. According to the national census of 2001, for the first time in the UK there are more people over age 60 years than under age 16 years [3]. However, a large proportion of people over age 60 suffer from chronic illnesses or disabilities [3,4]. In contrast to the UK, in some other countries (e.g. Japan) it has been found that extended lifespan does not necessarily translate into increased morbidity [3,4].

Coping with the impacts of these demographic changes is one of the greatest challenges for the 21^st ^century. However, ageing research in the UK is largely fragmented, underrepresented, poorly coordinated and lacks adequate funding support. In 2005, The House of Lords Select Committee on Science and Technology undertook an inquiry, chaired by Lord Sutherland of Houndwood, to evaluate the scientific aspects and current situation of ageing research in the UK. Among other findings, the inquiry reported "the attempts at coordination so far made under the aegis of the research councils are woefully inadequate. The image we have is of a series of ill-thought-out initiatives which have long titles, short lives, vague terms of reference, little infrastructure, and no sense of purpose. A radical reorganisation is essential" [5]. This inadequacy identified by the enquiry and the current demographic changes were the primary incentives for convening a 'Spark Workshop' -a two-day meeting, in which researchers from different areas across industry, charities, government and academia, share knowledge, explore potential novel concepts, and assess possibilities for future activities. The aims of the workshop were (i) to evaluate the future of ageing research in the UK, and (ii) to formulate a vision for action.

The workshop, held in May 2006, was jointly convened by the Funders' Forum for Research on Ageing and Older People (FFRAOP, or Funders' Forum) – a multi-agency alliance with a remit of coordinating ageing research in the UK- and the Corporate Research division of Unilever -a multinational foods, home and personal care company with a longstanding interest in promoting healthy ageing.

## What is ageing and ageing research?

Ageing is not a disease; it is a multi-factorial process that can be defined as the progressive loss of function accompanied by increasing morbidity and decreasing fertility with advancing age [6]. However, ageing is often perceived clinically as a collection of diseases [7]. Confusion about the precise nature of the relation between ageing and disease is a longstanding issue that has impeded the necessary progress towards the understanding of the intrinsic ageing process [7]. Ageing research must be independently recognized and funded to gain a clear understanding of the mechanisms that provoke the preceding vulnerability to the development of age-related disorders.

**Figure 1:**
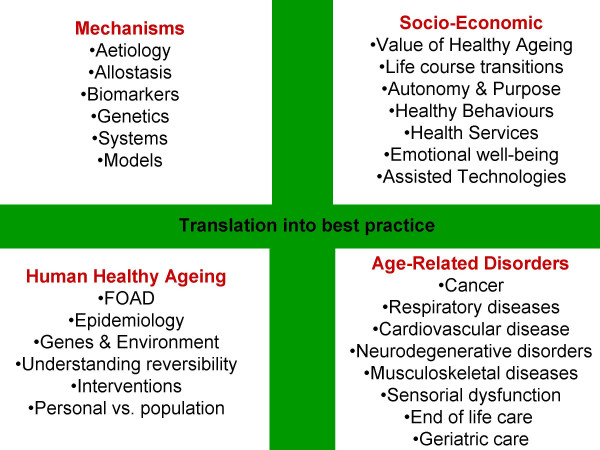
**Areas of ageing research**. Abbreviations: FOAD, Fetal Origins of Adult Disease.

Ageing research can be classified into different areas: (i) the mechanisms of ageing, (ii) the socio-economic factors of ageing, (iii) research aimed to achieve healthy ageing in humans and (iv) research on age-related disorders (Figure 1). All must be considered and supported, in order to achieve effective ways to arrive at best-practice routes to extend healthy life expectancy. But at present there is an important imbalance in the amount of resources destined to subsidise research initiatives associated with age-related disorders rather than funding all the areas of ageing research, particularly healthy ageing.

## What is the situation of ageing research in the UK?

**Figure 2:**
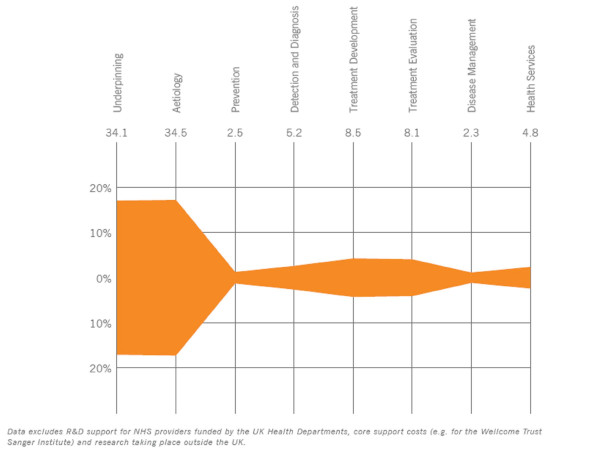
Proportion of combined research activity, Kite diagram*. *Source: UK Clinical Research Collaboration. UK Health Research Analysis. 2006 Available from:

In the UK the research emphasis on end-point disease has lead to a situation where relatively few resources are allocated to support ageing research [5]. In the recently published UK Health Research analysis -an effort to map the current UK research portfolio- ageing research is not separately identified [8]. Moreover, research in prevention -including preventable age-related disorders- comprises no more that 2.5% of all health-related research (Figure 2) [8].

Not only are resources limited, but there is also a grave lack of the coordination and direction needed to scope and secure the missing funds [5]. After a not very successful attempt by the National Collaboration on Ageing Research (NCAR) established in 2001 and dismantled in 2005, the Funders' Forum (FFRAOP) is currently the sole organization with the role of representing and coordinating British ageing research [5]. To compound matters, the Forum is not mandated to direct research, has no dedicated corporate budget and has to proceed on the basis of consensus between its members.

## Longevity: cost or gain?

Throughout the 19^th ^and 20^th ^centuries, the UK has experienced a sustainable increase in longevity, which is still ongoing. Although few would argue that the postponement of death is a tangible gain, the value of increasing longevity is difficult to translate into monetary terms, and there has often been an undue focus on the perceived negatives (health and social care costs, including pensions). However, using individual's willingness to pay for improvements that statistically extend life expectancy, Murphy and Topel have devised a framework for valuing the gains in health and longevity that allows calculating the economic gains from past, present and prospective future improvements in longevity and mortality rates [9]. When applied to the situation seen in the United States during the 20^th ^century, the authors found that the overall economic gains of increased longevity are enormous. Gains in life expectancy over the century were worth over $1.2 million per person to the current US population. From 1970 to 2000, gains in life expectancy added about $3.2 trillion per year to national wealth. In the UK these numbers can be expected to be proportionally similar as the longevity gains have been comparable [1]. It may be inferred from these analyses that gains in life expectancy directly translate into economic gains for individuals and society as a whole. As research-led advances in biomedicine are an important source of gains in longevity, investments in research on ageing should properly be seen within the context of their potentially enormous societal returns.

## Increased ageing society = increased productivity?

Increased longevity, if accompanied by increased health span, may mean that significant numbers of the labour force reaching a fixed retirement age will still have a full or considerable capacity for production. Furthermore, it has been found that age of retirement has a significant influence in remaining life expectancy [10]. Tsai SP et al reported an improvement of mortality with increasing age at retirement independent of socioeconomic status. Life expectancy for those retiring at age 55 was found to be 5 years shorter compared to those that retired at age 65 [10]. However, further research is necessary to identify the factors and mechanisms associated with an increased life expectancy after late retirement.

It is well known that work appears to be an important part of an individual's life providing a role and status in society and structure to the daily routine. These findings support current initiatives from different institutions and governments promoting policy changes to increase the current age of retirement. Thus, if ageing research can deliver healthier, longer lives, it can also deliver increased wealth by permitting extended social and economic productivity.

## Science of ageing: should it be multidisciplinary?

Ageing is a complex process influencing multiple mechanisms and physiological systems. Therefore it is critical that ageing research involves multidisciplinary research teams following a complementary approach. Furthermore, ageing has to be contextualised in terms of the social, economic and physical environments in which it takes place. New research initiatives in the ageing field could help to identify factors that characterise individuals that reach very old ages in good health. In this context it is particularly important to define and identify the 'healthy phenotype' as well as detect early deviations from this state, in order to permit intervention when a condition is still reversible. These factors can be of different order, including environmental, social, lifestyle and genetic factors; and they act together at different levels: intrinsically and extrinsically, to modify the human life course. Much remains unknown about how intrinsic and extrinsic factors interact to affect longevity and the way these mechanisms might be influenced to reach the ultimate goal of ageing research: increase quality and length of life.

## Is age-related disease reversible (the example of CVD)?

Cardiovascular disease (CVD) prevention is particularly important in preventing age-related morbidities. Ageing has been shown to affect the structure of the arterial wall allowing for infiltration and retention of oxidised lipids with associated chronic inflammation [11-13]. However, this is not an inevitable process; it is likely that age-associated arterial changes can be attenuated or delayed. Certain lifestyle modifications and medications have shown a potential reversibility of the age-related arterial changes that lead to CVD [11,13,14]. But, whether early attenuation of pathological arterial ageing will prevent or delay clinical CVD still needs to be directly evaluated. New efforts on ageing research should focus on identifying the factors and mechanisms that could affect retardation and perhaps reversal of the early pathological process, as well as effective interventions to positively modify these factors and prevent or delay CVD.

## Can we beat cognitive decline?

Ageing is also related to progressive cognitive decline [15-17]. However, as with arterial ageing, severe cognitive decline does not appear to be an inevitable process [15-17]. Alzheimer disease (AD), one of the most relevant and frequent causes of cognitive decline, is only genetically determined to a small extent, and modifiable environmental and lifestyle factors (e.g. smoking, nutrition, physical inactivity and low social activity) are thought to play a key role in its development [15-17]. New research should aim to identify the factors linked with cognitive decline and to clarify the ways environment and genes interact to foment neurological deterioration.

## Is there a way to change our disabling environments?

Besides CVD and cognitive decline, arthritis and sensorial dysfunction are conditions that although not related with a large mortality impact, are highly related with ageing and amply affect the capacity of the elderly population to interact with their surrounding environment. Due to the high prevalence of these limiting conditions among the elderly and to the fact that many of them are less acquainted with the state of the art in technology, elderly populations find it particularly challenging to keep up with the latest technological advances. It is fundamental for today's society to consider the needs and limitations of a progressively growing elderly population in the generation and design of new technology. To this end it is vital to create an environment where elderly individuals are empowered to live independently as well as to interact and communicate with a minimum level of limitation. Information technology, assistive technology and inclusive design are all essential tools to facilitate a society fit for older people [18,19]. These disciplines can effectively minimise the technological impact and limitations imposed by progressive developments by more fully involving functionally-limited older people in the research process [18-20]. These tools however, are not commonly implemented in the construction and design of products, homes, urban environment, public buildings and transportation systems. Increasing the funds for technology research on health, social and housing services, and involving the elderly in the design and production processes, will aid to eliminate disabling environments for disabled people.

## What defines quality of life for older people?

Ageing research should focus on aiding populations not only to live longer but also healthier and at a satisfactory level of quality of life (QoL) [21,22]. Quality of life requires a multidimensional parameter evaluation, using both intrapersonal and social-normative criteria, and taking account of an individual in time (past, current and anticipated). Great differences exist at inter- and intra-personal levels in the assessment of QoL. Influential variables on QoL include: marital status, income, health status and social environment [22]. Designing and implementing interventions to positively modify some of these factors may guarantee a better quality of life for the elderly by for example facilitating social participation, supporting social networks, employing older workers and taking strong actions to eradicate ageism [22].

On the other hand, QoL may act as a predictor *per se *of age-related disorders (e.g. CVD, AD), but research in this area is scattered and scarce. To clarify potential mechanisms involved in healthy ageing it is critical to discern the effect of QoL in longevity, morbidity and vice versa.

## Genetics of longevity: eating for a longer life?

Many epidemiological studies have found significant associations between diet quality and prevention of chronic disease and mortality [23]. Paradoxically, some nutrition intervention studies have failed to show benefits for ingredients (as opposed to foods) -e.g. anti-oxidant vitamins- for which, based on cellular studies in vivo, benefits had been expected [24,25]. It is clear that effects of putative agents differ by gender and ethnicity confirming a potential influence of genetic factors. For instance, several lipid candidate genes that have been explored in terms of CVD risk and longevity have shown significant gene-diet-environment interactions [26,27]. Hence, genetics is a key tool for future studies of mechanisms of age-related conditions. However, most of the studies in this area have been limited by their design (i.e. cross-sectional design, small sample size, limited SNP (Single Nucleotide Polymorphism) coverage of small candidate genes, and poor accountability of environmental factors). To benefit fully from the contribution of genetics, large prospective studies need to be designed, fully supported by extensive genotyping and analytical capacities. Furthermore, reliable intermediate phenotypes for ageing are urgently needed, both for genetic studies and for potential interventions.

## How to make it happen?

### The 10 commandments for ageing research in the UK

It is urgent that the UK adopts an integrated, multidisciplinary approach to ageing research, which recognizes its great importance as a societal and governmental priority. As it is identified in the House of Lords' report, the science of ageing in the UK is unstructured, under-funded and poorly coordinated. Since the 'ownership' of ageing science in the U.K. is at present dispersed and ambiguous, it is essential to re-structure and organize ageing research under a clear representation that could secure the funds needed to guarantee its full development. The efforts made for instance in Canada in the last few years, suggest that the creation of a 'virtual' ageing institute may contribute to create a stronger ownership and focus on ageing research, facilitate a multi-disciplinary approach to ageing science and assist young scientists to identify and develop career opportunities in the field. However, independent to the model adopted, it is evident that ageing research in the UK urgently requires a clearly defined organisational structure to guarantee its successful implementation and the realisation of its main goal: a population living longer, healthier and happier.

These and other conclusions reached during this 'Spark Workshop' have been summarized in a list of ten points, which constitute the backbone of a vision for action that could help to guarantee the successful implementation of ageing research in the UK:

1. Ageing is a highly differentiated and malleable process. Therefore, the commitment must be to develop interventions that can affect the ageing process or the experience of ageing in order to extend healthy life expectancy, independence and well-being in old age.

2. Investments in ageing research should be significantly increased as they are likely to produce immense gains to both the economy and society, in particular to the quality of life, productivity and self-sufficiency of the rapidly growing older population group

3. Society must recognise that improving the quality of life (QOL), of older people, including the promotion of active ageing and the eradication of ageism, is one of the biggest challenges of the 21^st ^century. This should translate into an integrated governmental policy for research on ageing as a key driver of QOL improvements.

4. Ageing research should reflect the complexity of the ageing process and integrate different dimensions of research into human healthy ageing, including the biological mechanisms and the socio-economic, cultural and psychological determinants of the ageing process

5. Healthy ageing research should concentrate on early, reversible stages of pathological conditions. As many lifestyle-related chronic diseases share common pathways of early dysregulation (e.g. CVD, AD), the development of markers, diagnostic techniques and interventions that can be applied to prevent late stage disease is fundamental

6. Ageing research should build on and expand existing longitudinal cohorts. These are critical to understand longevity and must combine genetic, socio-demographic and environmental aspects. It is crucial that future efforts embrace the role of genetics in ageing research given the variability of responses of individuals to drugs, nutrients and lifestyles due to different polymorphisms

7. Ageing research should pursue 'best practice' early interventions by creating an evidence base for translation to society, by engaging directly with its end users and, in particular, by ensuring that older people are a key reference point.

8. Research to inform the development and uptake of information technology, assistive technology and inclusive design must be implemented in the construction and design of products, homes, urban environments, public buildings and transportation systems to eradicate potentially disabling environments to functionally-limited older populations

9. The void in clear leadership, funding and representation of ageing research in the UK must be addressed. In particular, additional resources must be allocated to under-funded areas of ageing research (e.g. healthy ageing) to complement existing commitments to research aimed towards end-point chronic disease

10. It is critical that an overall ageing research portfolio is managed as a single entity across the contributing disciplines, which individually and collectively enhance understanding about the determinants and interventions that affect active ageing.

## Competing interests

The author(s) declare that they have no competing interests.

## Authors' contributions

All authors mentioned participated actively in all and each of the following aspects for this article:

• Conception and design, or analysis and interpretation of data

• Drafting the article or revising it critically for important intellectual content

• And final approval of the version to be published

## Pre-publication history

The pre-publication history for this paper can be accessed here:
